# Normothermic Crystalloid Polarizing Cardioplegia Improves Systolic and Diastolic Function in a Porcine Model of Cardiopulmonary Bypass

**DOI:** 10.3390/biomedicines13010070

**Published:** 2024-12-31

**Authors:** David Santer, Stefan Heber, Anne-Margarethe Kramer, Judith Radloff, Katharina Heissl, Attila Kiss, David J. Chambers, Seth Hallström, Bruno K. Podesser

**Affiliations:** 1 Ludwig Boltzmann Institute for Cardiovascular Research, Center for Biomedical Research and Translational Surgery, Medical University of Vienna, 1090 Vienna, Austria; david.santer@meduniwien.ac.at (D.S.); judith.radloff@meduniwien.ac.at (J.R.); katharina.heissl@meduniwien.ac.at (K.H.); attila.kiss@meduniwien.ac.at (A.K.); 2 Medical Faculty, University of Basel, 4001 Basel, Switzerland; 3 Institute of Physiology, Center for Physiology and Pharmacology, Medical University of Vienna, 1090 Vienna, Austria; stefan.heber@meduniwien.ac.at; 4 Clinical Department of Internal Medicine II—Cardiology, Nephrology, Intensive Care Medicine, University Hospital Wiener Neustadt, 2700 Wiener Neustadt, Austria; 5 Cardiac Surgical Research, The Rayne Institute (King’s College London), Guy’s and St Thomas’ NHS Foundation Trust, St Thomas’ Hospital, London SE1 7EH, UK; david.chambers@kcl.ac.uk; 6 Division of Medicinal Chemistry Otto Loewi Research Center, Medical University of Graz, 8010 Graz, Austria; seth.hallstroem@medunigraz.at

**Keywords:** cardioplegia, polarized arrest, depolarized arrest, normothermia, ischemia-reperfusion, cardioprotection

## Abstract

Background/Objectives: Previously, we showed that blood-based polarizing cardioplegia exerted beneficial cardioprotection during hypothermic ischemia; however, these positive effects of blood-based polarizing cardioplegia were reduced during normothermic ischemia compared to blood-based hyperkalemic (depolarizing) cardioplegia. This study compares crystalloid polarizing cardioplegia to crystalloid depolarizing cardioplegia in a normothermic porcine model of cardiopulmonary bypass; Methods: Twelve pigs were randomized to receive either normothermic polarizing (*n* = 7) or depolarizing (*n* = 5) crystalloid cardioplegia. After the initiation of cardiopulmonary bypass, normothermic arrest (34 °C, 60 min) was followed by 60 min of on-pump and 90 min of off-pump reperfusion. Myocardial injury (arterial CK-MB), hemodynamic function, and the energy status of the hearts were measured; Results: The arterial release of CK-MB was comparable between groups (*p* = 0.78) during reperfusion. During 150 min of reperfusion, systolic left ventricular pressure (*p* = 0.01) and coronary flow (*p* = 0.009) were increased, and wedge pressure (*p* = 0.04) was decreased in the polarized group. Further hemodynamic parameters (cardiac output, stroke volume) and high-energy phosphate levels were similar between groups. The requirement for noradrenaline administration during reperfusion was significantly higher (*p* = 0.013) in the polarized group; Conclusions: Under normothermic conditions and despite a similar increase in levels of cardiac CK-MB, crystalloid polarizing cardioplegia protected systolic and diastolic cardiac function after 60 min of cardiac arrest. These results suggest beneficial effects for polarizing cardioplegia; clinical studies are required to confirm these effects.

## 1. Introduction

In cardiac surgery, the introduction of cardioplegic solutions in the mid-1970’s, together with the progress in cardiopulmonary bypass (CPB) technology, enabled the “safe” induction of extended ischemic cardiac arrest. This enabled more complex cardiac surgery to become possible. However, discussions continue about the optimal strategy for cardioplegic cardiac arrest (from hypothermia to normothermia, crystalloid to blood, and depolarizing to polarizing solutions), and will remain controversial due to the continuous change in patient demand. The St Thomas’ Hospital cardioplegic solution utilizes moderately elevated potassium concentrations to induce a depolarized arrest; it has been extensively studied and is widely used [1]. Although blood-based depolarizing cardioplegic solutions have been shown to improve protection, and hence myocardial recovery, by attenuation of ischemic damage during cardiac arrest [2,3], overall mortality is not changed when compared to crystalloid cardioplegic solutions, and these continue to be used in many cardiac surgery centers. Nevertheless, depolarized arrest potentially causes contractile dysfunction and cell death by the intracellular accumulation of sodium and calcium [4]. Contractile dysfunction can lead to a failure to wean from CPB, necessitating the use of aortic balloon pumps or VA-ECMO to provide temporary support until recovery, thus highlighting the importance of effective cardioplegia.

As an alternative to depolarized arrest, we have demonstrated that an alternative concept of “polarized” arrest provides improved protection to the myocardium during global ischemia [5] and that esmolol, an ultra-short-acting beta-blocker can also induce polarized arrest by the inhibition of the fast sodium channel and the L-type calcium channel [6]. Our recent studies have expanded these concepts and shown that hypothermic polarizing blood cardioplegia, comprising esmolol, adenosine, and magnesium, improved hemodynamic recovery in pigs when compared to depolarizing blood cardioplegia [7]. In contrast, we also showed that normothermic polarizing blood cardioplegia led to reduced hemodynamic recovery and metabolic outcome [8]. One possible explanation is that red blood cell esterases rapidly metabolize the esmolol at tepid temperature into methanol and an acid metabolite [8]. Therefore, our subsequent aim was to test crystalloid polarizing cardioplegia under normothermic conditions. To further unravel the characteristics of polarizing cardioplegic solutions, the present study compares normothermic crystalloid polarizing versus conventional crystalloid depolarizing cardioplegia in a cardiopulmonary bypass (CPB) model in pigs. Based on the results of our previous studies [7,8], we hypothesized that normothermic crystalloid polarizing cardioplegia is superior to normothermic crystalloid depolarizing cardioplegia. Therefore, we assessed myocardial damage (CK-MB release), hemodynamic function, and myocardial high-energy phosphate levels during reperfusion.

## 2. Materials and Methods

### 2.1. Animals

Twelve female pigs (*Austrian Landrace*) were included in the study and randomized into two groups: crystalloid polarizing cardioplegia (POL, *n* = 7) and crystalloid depolarizing cardioplegia (DEPOL, *n* = 5; one additional animal in this group was excluded due to technical difficulties during reperfusion). Animal housing is described in Appendix A.

### 2.2. Protocol

**Figure 1:**
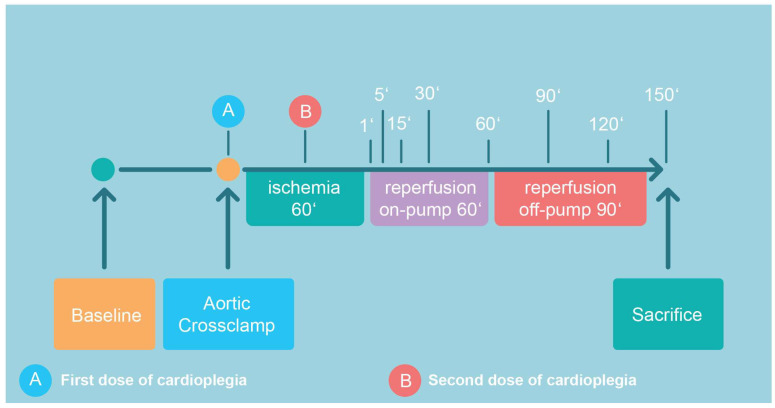
Following recording of baseline hemodynamics, cardiopulmonary bypass was initiated, aortic cross-clamping was performed with consecutive administration of the first dose of cardioplegic solution. The second dose was given after 30 min of ischemia (total ischemia time: 60 min). Aortic declamping was followed by a 60 min period of on-pump reperfusion, which included CPB weaning. After another period of 90 min of off-pump reperfusion, the animal was sacrificed. The indicated time points correspond to sampling points.

The detailed surgical CPB protocol is given in Appendix A. According to the protocol (Figure 1), all pigs were subjected to 60 min of ischemia, 60 min on-pump reperfusion, and another 90 min of off-pump reperfusion after decannulation and the administration of protamine (300 IU/kg). Prior to sacrifice (pentobarbital 300 mg/kg i.v.), samples from the anterior wall of the left ventricle were harvested for the analysis of high-energy phosphates (HEP; Figure 1).

### 2.3. Cardioplegic Solutions

The cardioplegic solutions used in this study were based on a cardioplegic solution containing esmolol, adenosine, and magnesium (POL) or modifications of the St Thomas’ Hospital cardioplegic solution No. 2 (DEPOL). DEPOL (Na^+^: 110.0 mmol/L, K^+^: 16.0 mmol/L, Mg^2+^: 16.0 mmol/L; Ca^2+^: 1.2 mmol/L) was provided as a 1000 mL solution by the hospital pharmacy of the General Hospital Linz. The basic composition of POL was: 1000 mL of Ringer’s solution with 1.0 mmol/L esmolol (Baxter, Vienna, Austria), 0.5 mmol/L adenosine (Sigma Aldrich, St. Louis, MO, USA), and 10.0 mmol/L magnesium gluconate (G.L. Pharma GmbH, Lannach, Austria). After aortic cross clamping, 1000 mL of the respective cardioplegic solution was infused with a pressure of 60 mmHg and a temperature of 34 °C via the aortic root; after 30 min of ischemia, an additional 500 mL of the cardioplegic solutions was infused. The final molar concentrations of both cardioplegic solutions are presented in Table 1.

### 2.4. Biochemical Analyses

Arterial blood samples were drawn at baseline (prior to CPB) and 1, 5, 15, 30, 60, 90, 120, and 150 min of reperfusion. During CPB, venous samples were drawn from the coronary sinus during controlled on-pump reperfusion: baseline, 1, 5, 15, 30, and 60 min of reperfusion. An immunoassay was performed for the primary outcome parameter CK-MB (Cobas immunoassay CKL, ID 0-324, Roche, Germany). Assessment of the high-energy phosphate status is provided in Appendix A.

### 2.5. Hemodynamic Evaluation

The details of the hemodynamic variables measured are provided in Appendix A.

### 2.6. Statistical Analysis

Graphs were generated with GraphPad Prism (9.4.0, GraphPad Software, La Jolla, CA, USA), and IBM SPSS Statistics 27 (IBM Corporation, New York, NY, USA) was used for statistical analyses. Regarding the dependent variables, mixed linear models were utilized. First, data were visually inspected regarding relevant deviations from normal distribution and heteroscedasticity at all time points, and right-skewed data were log10-transformed. Data that follow an approximately normal distribution data are presented as mean and standard deviation, whereas right-skewed data are given as geometric mean with 95% confidence interval. “Group” was defined as a fixed between-subjects factor with levels of POL and DEPOL. “Time” was designated as the levels of a fixed within-subjects factor. Each animal was included as a level of a random factor. Baseline values were used as covariates to adjust the models for pre-treatment differences among animals. The restricted maximum likelihood (REML) method served for model estimation. A time by group interaction was tested based on appropriate covariance structures determined by the smallest Akaike information criterion value. If a significant interaction was tested, group differences were estimated by contrasts at each time point. If not, the interaction term was omitted, and the main effect of “group” was interpreted as the group difference applicable to all time points. All reported *p*-values are from two-sided tests, with *p*-values less than 0.05 considered significant. As marker for myocardial damage, arterial CK-MB release was defined as the primary endpoint as in our previous manuscripts [7,8]. All secondary outcome measures were not adjusted for multiplicity due to the exploratory nature of this study and have to be interpreted accordingly.

In order to maintain consistency with our previous manuscript [8], the number of cases was also calculated accordingly. We defined a minimally relevant group difference of 25%. With a power of 80%, accepting the probability of a type I error of 5%, 5 animals per group were needed.

## 3. Results

### 3.1. Baseline Characteristics

Animals in both groups showed similar body weights (POL: *n* = 7, 48 ± 9 kg; DEPOL: *n* = 5, 49 ± 10 kg) and heart weights (POL: 296 ± 57 g; DEPOL: 236 ± 37 g).

### 3.2. Description of Cardioplegic Arrest

Time to asystole was longer in the POL group compared to the DEPOL group (POL: 234 ± 78 s; DEPOL: 156 ± 114 s; *p* = 0.21) but these differences were not significant. Six (out of seven) animals in the POL group and three (out of five) in the DEPOL group showed ventricular fibrillation (VF) during the first minutes of reperfusion (*p* = 0.22). In the POL group, VF was observed in two animals during ischemia, leading to an earlier administration of the second dose of cardioplegia. Temporary pacing was required for three (out of five) animals in the DEPOL group, whereas no pacing was required in the POL group (*p* = 0.11). Although a higher number of DC shocks were needed to induce sinus rhythm in the POL group, the difference was not statistically significant (POL: 2.1 ± 0.6; DEPOL: 0.8 ± 0.3; *p* = 0.13)

### 3.3. Myocardial Damage

**Figure 2:**
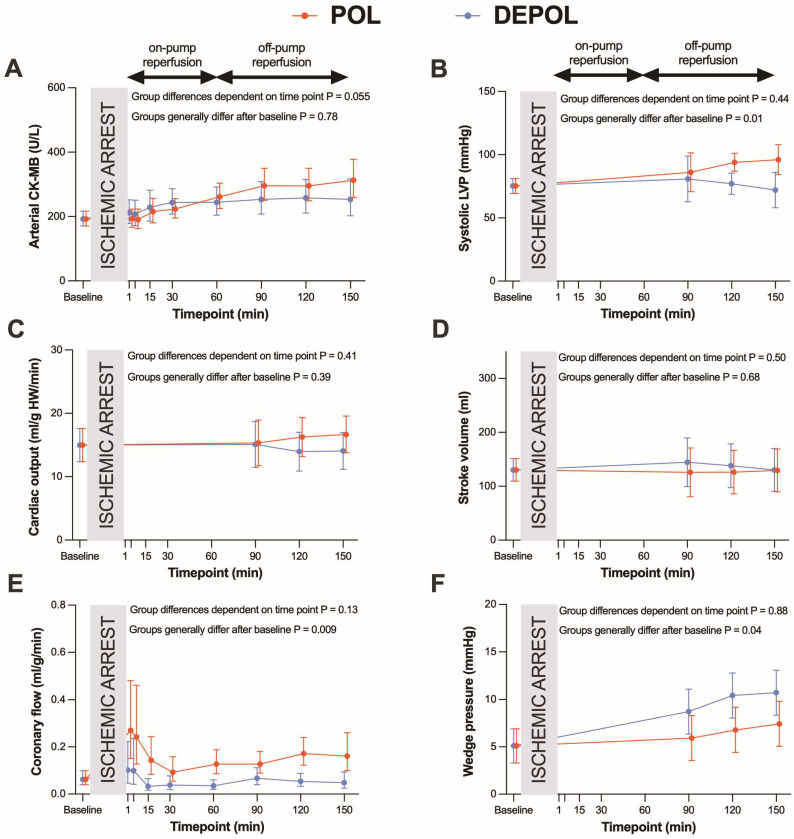
Effects of POL (red lines) and DEPOL (blue lines) on the primary outcome parameter arterial CK-MB and secondary outcome parameters. (**A**) There was no relevant difference between POL and DEPOL at all time points (*p* = 0.78). (**B**) Systolic left ventricular pressure was higher in POL (*p* = 0.01). (**C**) Cardiac output was comparable in both groups (*p* = 0.07). (**D**) The POL group showed markedly increased coronary flow (*p* = 0.29). (**E**) POL resulted in lower pulmonary capillary wedge pressure (*p* = 0.04). (**F**) Stroke volume was not different between POL and DEPOL (*p* = 0.68). On-pump reperfusion: time points 1–60 min; off-pump reperfusion: time points 90–150 min. Arithmetic or geometric means (depending on whether data were log-transformed for analysis) with 95% confidence intervals estimated by a mixed linear model that adjusts for baseline differences were used for the illustrations.

**Figure 3:**
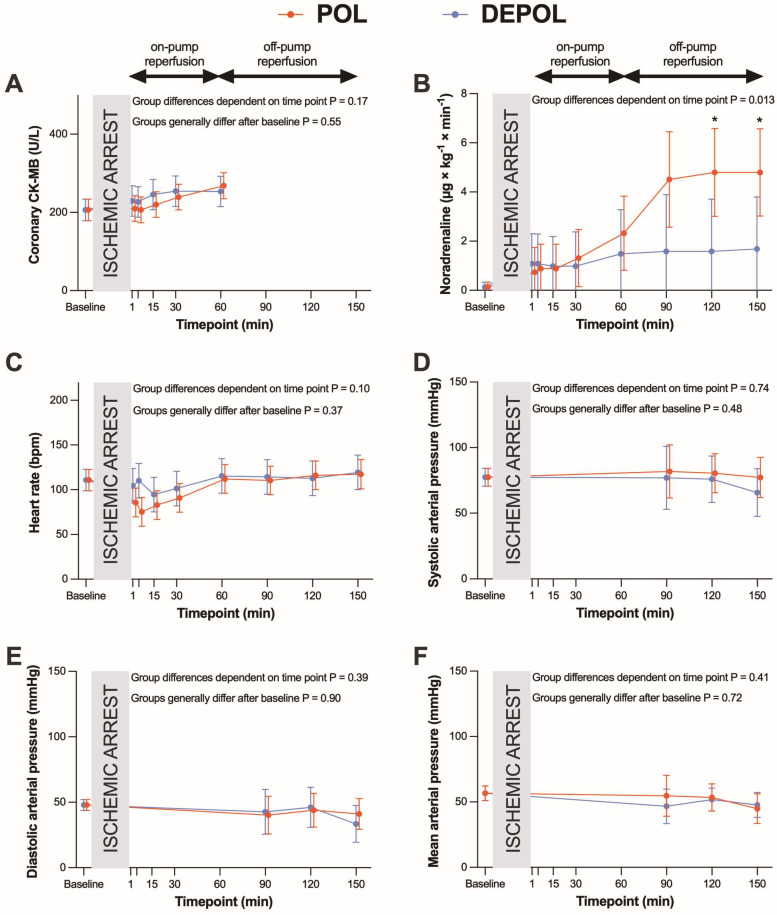
Effects of polarizing (POL, red lines) and depolarizing (DEPOL, blue lines) cardioplegic solutions on secondary outcome parameters. (**A**) No significant difference between POL and DEPOL in terms of coronary CK-MB at all time points (*p* = 0.55). (**B**) In the POL group, a higher requirement for noradrenaline was observed (*p* = 0.013). (**C**–**F**): There was no impact on heart rate (*p* = 0.37), APsys (*p* = 0.48), APdia (*p* = 0.90), or APmean (*p* = 0.72) by different cardioplegic solutions. Arithmetic or geometric means (depending on whether data were log-transformed for analysis) with 95% confidence intervals estimated using a mixed linear model that adjusts for baseline differences were used for the illustrations. On-pump reperfusion: time points from 1 to 60 min. Off-pump reperfusion: time points from 90 to 150 min. * *p* < 0.05.

The primary outcome parameter was the analysis of arterial CK-MB as a measure of myocardial cell damage. The release of arterial (*p* = 0.78; Figure 2A) and coronary CK-MB (*p* = 0.55, Figure 3A) after ischemia was similar in both groups.

### 3.4. Hemodynamics

The secondary outcomes were parameters of hemodynamic function. Systolic left ventricular pressure was higher (*p* = 0.01, Figure 2B) at the end of reperfusion in POL, whereas cardiac output (CO, *p* = 0.39; Figure 2C) and stroke volume (SV, *p* = 0.68, Figure 2D) were comparable, and coronary flow (CF, *p* = 0.009; Figure 2E) and wedge pressure (*p* = 0.04; Figure 2F) were markedly improved in POL.

These differences were observed at the time points of 90, 120, and 150 min. The POL group showed a significantly increased requirement for noradrenaline from 120 to 150 min (*p* = 0.013; Figure 3B). Heart rate (HR), systolic arterial pressure (APsys), mean arterial pressure (APmean), and diastolic arterial pressure (APdia) showed no significant changes in either group (Figure 3C–F).

### 3.5. High-Energy Phosphates

**Figure 4:**
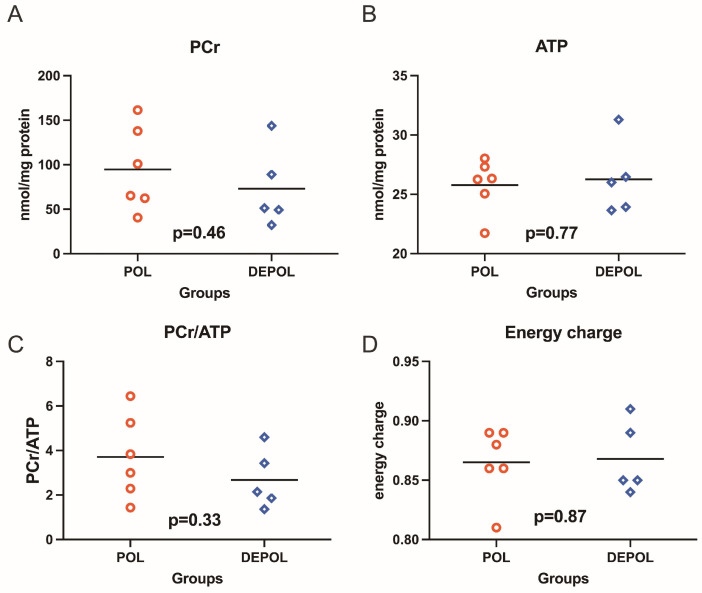
High-energy phosphates, including phosphocreatine (PCr), adenosine triphosphate (ATP), as well as PCr/ATP ratio and energy charge were determined from freeze-clamped left ventricular biopsies obtained immediately after 150 min of reperfusion. The levels of PCr and ATP showed similar preservation across both groups (**A**,**B**). No relevant differences were observed in the PCr/ATP ratio (**C**) or energy charge (**D**). POL: polarizing cardioplegic solution; DEPOL: depolarizing cardioplegic solution.

The analysis of high-energy phosphate levels showed a similar preservation concerning phosphocreatine (PCr), adenosine triphosphate (ATP), PCr/ATP ratio, and energy charge in both groups (Figure 4).

## 4. Discussion

Our previous study examining normothermic blood-based polarizing cardioplegia suggested that the cardioprotective efficacy may be compromised, especially if esmolol is added to blood prior to the use of the cardioplegia. This is potentially due to the rapid degradation of esmolol by red blood cell esterases [8]. This degradation could be avoided by using a crystalloid esmolol cardioplegic solution, or by adding the crystalloid solution to the blood vehicle immediately prior to use. Therefore, in the present study of polarized arrest, we chose to use a normothermic crystalloid polarizing solution and to compare it to a conventional crystalloid depolarizing cardioplegic solution in a CPB model in pigs. Myocardial damage (indicated by release of arterial CK-MB) was defined as the primary outcome. The present study revealed the following: (i) myocardial damage was comparable in POL and DEPOL groups; (ii) contractility (systolic LVP, wedge pressure) and CF was significantly improved in POL.

Myocardial protection has been shown to be improved by the addition of 1 mmol/L of adenosine to a depolarizing blood cardioplegic solution in patients undergoing heart valve surgery [9]; cardiac troponin-I and IL-8 were lower at 10 min and IL-6 was lower 24 h after aortic declamping. In another study, the continuous coronary infusion of warm esmolol-enriched blood during cardiopulmonary bypass was evaluated in 41 patients undergoing aortic valve surgery [10]. Esmolol successfully reduced transmyocardial oxygen content but had no influence on hemodynamics or plasma troponin-I levels. In our recent studies in pigs, both normothermic [8] as well as hypothermic [7] blood-based polarized cardioplegic arrest demonstrated comparable myocardial damage (arterial CK-MB) when compared to the depolarizing cardioplegic solution. CK-MB release is a less sensitive marker of myocardial injury than troponin, and hence may explain why small, but significant, differences in hemodynamic recovery did not reflect differences in CK-MB levels post-operatively. Since high-energy phosphate levels were similar between the POL and DEPOL groups under normothermic crystalloid conditions, esmolol degradation might have been decelerated due to the lack of warm blood. As a consequence, the cardioprotective effects of esmolol have also been shown in other normothermic settings [11]. The present normothermic crystalloid data again confirm the cardioprotective potential of polarized arrest. We interpret these results as promising since even after decades, the control solution (depolarizing St Thomas’ No. 2) is still commonly used in clinical practice, provides excellent myocardial protection, and serves as a benchmark in experimental and clinical studies [12].

The design of this current study was based on previous results [8], which had shown that normothermic blood-based polarizing cardioplegia was inferior to depolarizing blood-based cardioplegia in pigs in terms of hemodynamic and metabolic performance. Our explanation for this inferiority was that the ultra-short-acting beta-blocker esmolol was metabolized by red blood cell esterases [13], and therefore, the efficacy of this normothermic blood composition was reduced. In isolated rat hearts, polarizing arrest has been shown to be cardioprotective [14]. While the addition of esmolol to cold blood cardioplegia did not improve cardioprotection during urgent coronary revascularization in unstable patients [15], it has been shown to reduce myocardial oxygen metabolism [10] in hypertrophic ventricles and reduce postoperative inotropic demand [16] in cold blood solutions. The present study shows a slightly different picture; significantly improved systolic LVP and CF as well as reduced wedge pressures were observed during reperfusion, while noradrenaline demand was significantly elevated in POL. Experimental data have shown that esmolol has a lowering effect on left ventricular dp/dt [17] but not on CO in dogs. In patients with idiopathic dilated cardiomyopathy, treatment with esmolol increased coronary blood flow, while there was no change in healthy controls. A potential explanation is that esmolol decreases left ventricular wall stress [18] by the induction of coronary hyperemia. In addition, coronary artery diameter may be increased by beta-1 blockade with metoprolol [19]. In patients with acute myocardial ischemia, esmolol induced systolic arterial blood pressure reduction but had no effect on pulmonary wedge pressure [20,21]. Furthermore, the increased systolic LVP and CF can be explained by the high levels of noradrenaline, which has been shown to improve systolic LVP by coronary vasodilation [22].

In general, beta-blockers are not known for their hypotensive effects. However, esmolol is commonly used as an antihypertensive drug, but its hypotensive effect is not explained solely by beta-blockade [23]. Therefore, it is unlikely that the high demand for noradrenaline in the POL group during the reperfusion phase is caused by esmolol, which is an ultra-short-acting compound [24,25]. High doses of Mg^2+^ have been shown to reduce systemic arterial pressure and arterial vascular resistance in dogs [26]. Also known as a natural calcium channel blocker, Mg^2+^ has vasodilatory effects [27] and inhibits noradrenaline release by blocking N-type Ca^2+^ channels at nerve endings [28]. In this study, doses of noradrenaline were higher in POL to sustain mean blood pressure targets. The most obvious explanation for these side effects is the high Mg^2+^ concentration in combination with esmolol [29], which are distributed systemically after aortic declamping in POL.

### 4.1. Future Implications of Polarized Arrest

The invention of depolarizing cardioplegic solutions was crucial for the advancement of cardiac surgery. However, they may cause sodium and calcium overload, which can be particularly harmful to damaged hearts. Polarized arrest, administered as a cold blood-based solution, has shown benefits [7,30]. While we were able to characterize polarized arrest in a series of studies [7,8], a first-in-man study is needed to evaluate its true clinical potential. Nonetheless, polarizing cardioplegic solutions (Verona ALM: adenosine, lidocaine and Mg^2+^) have already demonstrated safety, efficacy, and superiority over Buckberg cardioplegia in a prospective randomized clinical study [31].

### 4.2. Limitations

Due to the relatively low number of animals per group, the estimates of group differences are subject to a certain degree of uncertainty; this should be considered when interpreting the results. Thereby, it has to be considered that the sample size calculation was based on a t-test for independent samples, as no reliable assumptions could be made prior to data collection regarding the covariance structure of the repeated measures in a mixed model or the correlation between baseline values and later measurement time points. However, in the actual analysis, we employed a mixed model that included a baseline covariate, which explained the variance of the later time points. This approach likely contributed to more robust results than initially anticipated. Nevertheless, it is important to emphasize that this is a preclinical study, and definitive conclusions can only be drawn from randomized controlled trials in humans. Another limitation is that assays to measure porcine troponin were not available to us at the time of the experiments and no blood samples were left for performing troponin analyses at the time of manuscript submission. Although cardiac troponin is the gold standard for acute myocardial injury [32], it is especially superior to CK-MB as a prognostic marker. In the Fourth Universal Definition of Myocardial Infarction, CK-MB was mentioned as inferior to cardiac troponin but still as a “good” parameter and CK-MB is recommended as best alternative to cardiac troponin [33].

## 5. Conclusions

Normothermic cardioplegic arrest with POL showed superior contractility and coronary flow compared to DEPOL, but no superiority in terms of cardiac enzymes or high-energy phosphates. The increased demand of vasoconstrictors in POL has to be observed in future studies. In conclusion, normothermic crystalloid polarizing cardioplegic solution provided safe cardioplegic arrest in this cardiopulmonary bypass model in pigs.

## Figures and Tables

**Table 1:** Molar concentrations of cardioplegic solutions.

Components	POL (*n* = 7)	DEPOL (*n* = 5)
Esmolol, mmol/L	1.0	-
Adenosine, mmol/L	0.5	-
Magnesium, mmol/L	10	16
Sodium, mmol/L	110	110
Potassium, mmol/L	4	16
Calcium, mmol/L	1.2	1.2

Final molar concentrations in low-dose cardioplegic solutions (POL and DEPOL). The basic composition of STH-Pol was esmolol, adenosine, and magnesium gluconate mixed in 1 L of Ringer’s solution. POL, polarizing cardioplegia; DEPOL, depolarizing cardioplegia.

## Data Availability

The data underlying this article will be shared on reasonable request to the corresponding author.

## Appendix A

### Appendix A.1. Housing

Twelve female *Austrian Landrace* pigs were housed under controlled conditions at our institution (Center for Biomedical Research, Medical University of Vienna, Vienna, Austria). The conditions were 12 h of light and dark, temperatures between 19 and 21 °C, and humidity levels of 40–60%. The pigs were fed a standard diet from Sniff GmbH, Soest, Germany, twice daily, with ad libitum access to water. Trained staff provided care in accordance with the Federation of European Laboratory Animal and Science Associations (FELASA) guidelines.

### Appendix A.2. Protocol

The pigs were anesthetized with premedication of ketamine (15 mg/kg i.m.) and acepromazine (1.3 mg/kg i.m.), followed by anesthesia with propofol (2.5 mg/kg), piritramid (15 mg), and rocuronium bromide (20 mg). Anesthesia maintenance included piritramid (1.875 mg/kg/h), rocuronium bromide (3.7 mg/kg/h), and propofol (10 mg/kg/h). The animals were intubated and ventilated. Invasive arterial pressure monitoring was conducted via the left femoral artery, and medication was administered through a central venous catheter in the left external jugular vein. Hemodynamic management involved volume substitution and noradrenaline to maintain a systolic blood pressure above 70 mmHg. Following sternotomy, heparin (300 IU/kg) was administered before cannulation for cardiopulmonary bypass (CPB), using an Optisite arterial cannula and a Trim Flex venous cannula (Edwards Lifesciences Corp, Irvine, CA, USA). Hemodynamic monitoring included measurements of cardiac output (CO), coronary flow (CF), left ventricular pressure (LVP), and coronary blood samples during the first 60 min of ischemia and reperfusion. Baseline parameters were recorded before starting CPB, cross-clamping the aorta, and administering the cardioplegic solution via antegrade perfusion.

### Appendix A.3. Biochemical Analyses

Energy status was assessed by measuring high-energy phosphate levels from tissue samples of the left ventricular (LV) mid-anterior wall. Freeze-clamped biopsies were stored in liquid nitrogen prior to analysis. A piece of frozen tissue (50–100 mg) was homogenized in 500 µL of 0.4 mol/L perchloric acid using PFTE shaking flasks pre-cooled in liquid nitrogen and a microdismembrator (Microdismembrator U Ball Mill; Satorius, Germany). After thawing and centrifugation at 12,000× *g*, 200 µL of the acid extract was neutralized with 20–25 µL of 2 mol/L potassium carbonate at 4 °C. The supernatant obtained after centrifugation (20 µL injection volume) was used for HPLC analysis. The pellets were dissolved in 1 mL of 0.1 mol/L sodium hydroxide and diluted 1:10 with physiologic saline for protein determination using a BCA protein assay. Adenosine triphosphate (ATP), adenosine diphosphate (ADP), adenosine monophosphate (AMP), and phosphocreatine (PCr) were measured by HPLC. Separation was performed on a Hypersil ODS column using a VWR Hitachi system. Detector signals were recorded at 214 nm (PCr) and 254 nm, and the EZchrom Elite program was used for data acquisition and analysis. Energy charge (EC) was calculated as: EC = (ATP + ½ ADP)/(ATP + ADP + AMP).

### Appendix A.4. Hemodynamic Evaluation

Secondary outcome parameters (including left ventricular systolic pressure (LVPsys), cardiac output (CO), stroke volume (SV), external heart work (EHW), coronary flow (CF), mean arterial pressure (APmean), diastolic and systolic arterial pressure (APsys, APdia), heart rate (HR), wedge pressure, and noradrenaline demand) were recorded as baseline (pre-CPB) and during reperfusion at 1, 5, 15, 30, 60, 90, 120, and 150 min. Cardiac output (mL/g) is presented as COabs divided by heart weight and multiplied by 1000. Stroke volume (mL/g/beat) was calculated using the formula COabs divided by heart rate divided by heart weight multiplied by 1000, and then multiplied by 1000 again. External heart work (mL/g) was assessed using the formula CO divided by heart weight multiplied by LVPsys. Coronary flow (mL/g/min) is shown as CF divided by heart weight.

## References

[1] Robinson L.A., Schwarz G.D., Goddard D.B., Fleming W.H., Galbraith T.A. (1995). Myocardial protection for acquired heart disease surgery: Results of a national survey. Ann. Thorac. Surg..

[2] Fremes S.E., Christakis G.T., Weisel R.D., Mickle D.A., Madonik M.M., Ivanov J., Harding R., Seawright S.J., Houle S., McLaughlin P.R. (1984). A clinical trial of blood and crystalloid cardioplegia. J. Thorac. Cardiovasc. Surg..

[3] Guru V., Omura J., Alghamdi A.A., Weisel R., Fremes S.E. (2006). Is blood superior to crystalloid cardioplegia? A meta-analysis of randomized clinical trials. Circulation.

[4] Chambers D.J., Fallouh H.B. (2010). Cardioplegia and cardiac surgery: Pharmacological arrest and cardioprotection during global ischemia and reperfusion. Pharmacol. Ther..

[5] Snabaitis A.K., Shattock M.J., Chambers D.J. (1997). Comparison of polarized and depolarized arrest in the isolated rat heart for long-term preservation. Circulation.

[6] Fallouh H.B., Bardswell S.C., McLatchie L.M., Shattock M.J., Chambers D.J., Kentish J.C. (2010). Esmolol cardioplegia: The cellular mechanism of diastolic arrest. Cardiovasc. Res..

[7] Santer D., Kramer A., Kiss A., Aumayr K., Hackl M., Heber S., Chambers D.J., Hallstrom S., Podesser B.K. (2019). St Thomas’ Hospital polarizing blood cardioplegia improves hemodynamic recovery in a porcine model of cardiopulmonary bypass. J. Thorac. Cardiovasc. Surg..

[8] Kramer A.M., Kiss A., Heber S., Chambers D.J., Hallstrom S., Pilz P.M., Podesser B.K., Santer D. (2022). Normothermic blood polarizing versus depolarizing cardioplegia in a porcine model of cardiopulmonary bypass. Interact. Cardiovasc. Thorac. Surg..

[9] Liu R., Xing J., Miao N., Li W., Liu W., Lai Y.Q., Luo Y., Ji B. (2009). The myocardial protective effect of adenosine as an adjunct to intermittent blood cardioplegia during open heart surgery. Eur. J. Cardio-Thorac. Surg. Off. J. Eur. Assoc. Cardio-Thorac. Surg..

[10] Scorsin M., Mebazaa A., Al Attar N., Medini B., Callebert J., Raffoul R., Ramadan R., Maillet J.M., Ruffenach A., Simoneau F. (2003). Efficacy of esmolol as a myocardial protective agent during continuous retrograde blood cardioplegia. J. Thorac. Cardiovasc. Surg..

[11] Liu X., Shao F., Yang L., Jia Y. (2016). A pilot study of perioperative esmolol for myocardial protection during on-pump cardiac surgery. Exp. Ther. Med..

[12] Reidy M.R., Jimenez E., Omer S., Cornwell L.D., Runbeck S.X., Preventza O., Loor G., Rosengart T.K., Coselli J.S. (2021). Single-Dose del Nido Cardioplegia Compared With Standard Cardioplegia During Coronary Artery Bypass Grafting at a Veterans Affairs Hospital. Tex. Heart Inst. J..

[13] Quon C.Y., Stampfli H.F. (1985). Biochemical properties of blood esmolol esterase. Drug Metab. Dispos..

[14] Nishina D., Chambers D.J. (2018). Efficacy of esmolol cardioplegia during hypothermic ischaemia. Eur. J. Cardio-Thorac. Surg. Off. J. Eur. Assoc. Cardio-Thorac. Surg..

[15] Rinne T., Harmoinen A., Kaukinen S. (2000). Esmolol cardioplegia in unstable coronary revascularisation patients. A randomised clinical trial. Acta Anaesthesiol. Scand..

[16] Zangrillo A., Bignami E., Noe B., Nardelli P., Licheri M., Gerli C., Crivellari M., Oriani A., Di Prima A.L., Fominskiy E. (2021). Esmolol in Cardiac Surgery: A Randomized Controlled Trial. J. Cardiothorac. Vasc. Anesth..

[17] Gorczynski R.J. (1985). Basic pharmacology of esmolol. Am. J. Cardiol..

[18] Skalidis E.I., Hamilos M.I., Chlouverakis G., Kochiadakis G.E., Parthenakis F.I., Vardas P.E. (2007). Acute effect of esmolol intravenously on coronary microcirculation in patients with idiopathic dilated cardiomyopathy. Am. J. Cardiol..

[19] Billinger M., Seiler C., Fleisch M., Eberli F.R., Meier B., Hess O.M. (2001). Do beta-adrenergic blocking agents increase coronary flow reserve?. J. Am. Coll. Cardiol..

[20] Krumpl G., Ulc I., Trebs M., Kadlecova P., Hodisch J. (2017). Bolus application of landiolol and esmolol: Comparison of the pharmacokinetic and pharmacodynamic profiles in a healthy Caucasian group. Eur. J. Clin. Pharmacol..

[21] Kirshenbaum J.M., Kloner R.A., Antman E.M., Braunwald E. (1985). Use of an ultra short-acting beta-blocker in patients with acute myocardial ischemia. Circulation.

[22] Vatner S.F., Higgins C.B., Braunwald E. (1974). Effects of norepinephrine on coronary circulation and left ventricular dynamics in the conscious dog. Circ. Res..

[23] Deegan R., Wood A.J. (1994). Beta-receptor antagonism does not fully explain esmolol-induced hypotension. Clin. Pharmacol. Ther..

[24] Byrd R.C., Sung R.J., Marks J., Parmley W.W. (1984). Safety and efficacy of esmolol (ASL-8052: An ultrashort-acting beta-adrenergic blocking agent) for control of ventricular rate in supraventricular tachycardias. J. Am. Coll. Cardiol..

[25] Cork R.C., Kramer T.H., Dreischmeier B., Behr S., DiNardo J.A. (1995). The effect of esmolol given during cardiopulmonary bypass. Anesth. Analg..

[26] Nakayama T., Nakayama H., Miyamoto M., Hamlin R.L. (1999). Hemodynamic and electrocardiographic effects of magnesium sulfate in healthy dogs. J. Vet. Intern. Med..

[27] Vickovic S., Pjevic M., Uvelin A., Pap D., Nikolic D., Lalic I. (2016). Magnesium Sulfate as an Adjuvant to Anesthesia in Patients with Arterial Hypertension. Acta Clin. Croat..

[28] Shimosawa T., Takano K., Ando K., Fujita T. (2004). Magnesium inhibits norepinephrine release by blocking N-type calcium channels at peripheral sympathetic nerve endings. Hypertension.

[29] Arar C., Colak A., Alagol A., Uzer S.S., Ege T., Turan N., Duran E., Pamukcu Z. (2007). The use of esmolol and magnesium to prevent haemodynamic responses to extubation after coronary artery grafting. Eur. J. Anaesthesiol..

[30] Aass T., Stangeland L., Chambers D.J., Hallstrom S., Rossmann C., Podesser B.K., Urban M., Nesheim K., Haaverstad R., Matre K. (2017). Myocardial energy metabolism and ultrastructure with polarizing and depolarizing cardioplegia in a porcine model of cardiopulmonary bypass. Eur. J. Cardio-Thorac. Surg. Off. J. Eur. Assoc. Cardio-Thorac. Surg..

[31] Onorati F., Dobson G.P., San Biagio L., Abbasciano R., Fanti D., Covajes C., Menon T., Gottin L., Biancari F., Mazzucco A. (2016). Superior Myocardial Protection Using “Polarizing” Adenosine, Lidocaine, and Mg^2+^ Cardioplegia in Humans. J. Am. Coll. Cardiol..

[32] Ruetzler K., Smilowitz N.R., Berger J.S., Devereaux P.J., Maron B.A., Newby L.K., de Jesus Perez V., Sessler D.I., Wijeysundera D.N., on behalf of the American Heart Association Council on Cardiopulmonary, Critical Care, Perioperative and Resuscitation (2021). Diagnosis and Management of Patients With Myocardial Injury After Noncardiac Surgery: A Scientific Statement From the American Heart Association. Circulation.

[33] Thygesen K., Alpert J.S., Jaffe A.S., Chaitman B.R., Bax J.J., Morrow D.A., White H.D. (2018). Fourth Universal Definition of Myocardial Infarction (2018). J. Am. Coll. Cardiol..

